# Fever

**Published:** 1828-05

**Authors:** 


[To be continued.]

---

### HOSPITAL REPORTS,

*(Principally condensed from various Periodical Publications.)*

### FEVER.

### *Cases of Fever, treated at the* INFIRMARY OF PHILADELPHIA.

THE following cases will serve to illustrate the practice of our transatlantic brethren in fever.

CASE I.—Moses Howard, (black,) aged twenty-three years, was admitted into the Almshouse Infirmary, on the evening of the 24th of September, 1826. He had been ill about three weeks. While at work at his trade, that of a blacksmith, he felt overcome with heat, and in this state he swallowed a potatoe, which caused immediate sickness of the stomach. Feeling thirsty, he took a drink of water, which was soon followed by puking, and he threw up what he had eaten at dinner. After vomiting he felt better. At dark he was worse, and had nausea. He rested tolerably well through the night. After this he was attacked with rigors every day, followed by fever, which set in at noon and continued through the afternoon and night. Towards morning there was a remission of the febrile paroxysm. For the last three days his fever had been continued, and was attended with delirium.

The symptoms on admission were—pulse quick, without tension; skin hot and dry; tongue foul, covered with a brown crust, except at the edges, dry, and rather contracted; lips dry, scaly; teeth fuliginous; eyes heavy, the upper half of the ball more injected than the inferior portion, suffused with tears. He has no appetite whatever; there is tension of the abdominal parietes; complains of pain in the abdomen, increased by pressure over almost every part of it. His head is painful, and attended with delirium.

25th.—This morning I find him about the same as last evening. He dressed himself, and was anxious to go down stairs; his hands are cold; he picks at the bedclothes, and has slight subsultus tendinum. I did not disturb him during the night, but commenced the treatment this morning as follows, viz.:

A blister to be applied to the occiput and back of the neck; bowels to be evacuated by simple injections; feet to be bathed in water as hot as can be endured. Diet to consist of preparations of sago; drink to consist of barley water, acidulated.

About eleven o'clock, Dr. Jackson saw the patient. At this time the eyeballs were highly injected and rolled upwards; skin cold; pulse small and frequent; stupor.

In addition to the treatment, four cups were directed to the head,—two of which were to be dry, and two with scarifications; also twelve leeches to the epigastrium, followed by warm fomentations to the abdomen, and frictions of terebinthinate decoction of Cantharides to the extremities.—The leeches were applied in the evening.—Blankets wrung out of hot water to his legs.

26th.—Very much improved. His strength has increased. There is reaction with more heat of the skin; a better pulse; no subsultus tendinum; eyes less injected, and not turned upwards.

28th.—Better.

Treatment consists in frequent application of blankets wrung out of hot water to his extremities. To have today a simple injection and a dose of Castor-oil. The warm fomentation to the abdomen to be continued. Injections of cold flaxseed mucilage.

29th.—Improving very fast: the heat of his skin is nearly natural; tongue becoming moist.

Diet today to have the addition of chicken water.

30th.—Improving.

The warm fomentations are still continued to the abdomen. Bowels kept open by simple injections of flaxseed tea.

October 1st.—Still improving; tongue nearly clean; pulse regular; strength increasing.

2d.—Sitting up this morning, but is ordered to bed, as he is not considered sufficiently recovered to bear exertion.

Diet—soup, gruel, &c.

Evening.—Complains that his bowels are confined. Ordered a simple injection to remove this.

3d.—Sitting up at times; complains yet of debility.

Diet—soup, bread, gruel, &c.; drink, barley water.—Directed him to drink of the following tonic decoction: ℞. Pulv. Cinchon. opt. ℥ss.; Rad. Colomb. ʒij.; Cortex Aurant. ʒj.; Cortex Cinnamon. ʒss. Add to a quart of boiling water; reduce by simmering to three half-pints.

7th.—Walking about; fairly convalescent.

23d.—This patient has rather a slow convalescence. This Dr. Jackson ascribes to too great an indulgence in eating meat, &c.; he therefore orders him to have nothing but barley water for twenty-four hours. To take also ℥ss. Ol. Ricini.

November 4th.—Convalescence proceeded rapidly under a more restricted regimen. Discharged cured.

*Observations.*—This case, at the time it was presented to our notice, offered a train of formidable symptoms. I remarked to the clinical class at the time, that it had been customary to combat the condition of the system, which was one of great prostration, with tonics and stimulants, freely administered. But this practice in the Almshouse having so frequently failed to accomplish the ends that had been looked for, I was discouraged in attempting it in the case then before us. The state of the intellectual faculties, the injected condition of the eye and its tendency to be constantly rolling upwards, with the convulsive twitchings of the muscles, were complete evidence to my mind that the meninges of the brain were the seat of considerable irritation: the dry, brown, parched tongue, the extreme sensibility of the epigastrium, the nausea that had early existed, were equal indications that the intermittent irritation had become fixed, and we had now a gastritis of no slight intensity to contend with. The prostration was, then, the consequence of this condition of the brain and stomach, by which, in the first place, the nervous functions were in a state of lesion, and were maintained in that state by the sympathetic irritation directed on the brain from the stomach; and, in the second place, the concentration of irritation in the stomach and small intestines, constituting them a focus of the vital energies and the circulating fluids, at the expense of other organs, was the immediate cause of the diminished actions and enfeebled functions of the skin, of the central organ of the circulation, and of the pulmonary system. From this view the indication was to diminish the excess of irritation in the brain and stomach, and operate a revulsion on the surface. Notwithstanding the degree of prostration and apparent debility, I would therefore venture on a direct but moderate local depletion, to act on the two points, the seats of the irritation; to allay that of the stomach by cooling and demulcent drinks; while the skin was excited by means of moist heat, by enveloping the half of the body in blankets wrung out of water as hot as could be borne, and renewed night and day as soon as they began to cool. This mode of stimulating the skin, when it is intended to produce a revulsion from the brain, I prefer to sinapisms or blisters, when cerebral or meningeal inflammation exists, as pain is a direct and energetic stimulus of the brain and almost every organ, and often aggravates, and even excites, inflammations in the brain, stomach, &c. The result of the treatment was highly gratifying, especially from the rapidity with which the very formidable symptoms disappeared. It is to be remarked that the convalescence was retarded by the full diet allowed, in the expectation of conferring strength.

Case II.—Ann Riggins, aged thirty, fair complexion, delicate frame, sober habits, admitted into the Almshouse, February 12th, 1826. She had been sick upwards of three weeks, and was a patient of one of the Dispensaries. After exposure to wet and cold, she had been seized with pains in the back, head, and extremities: fever succeeded, and which had continued until her entrance into the house. According to her statement, she had taken during her treatment four emetics, a number of powerful purgatives, and nitre powders. A large blister had been applied over the epigastrium, showing that gastric symptoms had existed, and her extremities were also blistered.

At her admission into the ward at twelve o'clock M. she presented the following symptoms: skin dry and husky, eyes injected, cheeks flushed; tongue dry, hard, cracked, pointed, brown, and crusted; bowels costive; extremities cold; pulse scarcely perceptible; breathing short and difficult; stupor amounting almost to insensibility; great distress evinced by pressing the epigastrium.

The lower half of the body was immediately wrapped in blankets wrung out of warm water; a common house injection was ordered. Acidulated barley water for drink.

Evening.—Skin more natural in temperature and feeling; pulse more developed; coma continues.

Cups to be applied to temples and back of neck. Cold to head. Warm fomentations to lower extremities to be continued during the night.—Same drink.

13th.—Is much better: stupor relieved; converses freely, and gave the history of her disease and previous treatment. Complains of no pain except when she coughs; no pain in head; eyes less injected, and face not as much flushed; tongue becoming moist, but is furred; feet warm; pulse full and soft; bowels freely opened by injection. Continue treatment.—Five P.M., slight increase of fever, with some difficulty of breathing; bowels not moved since morning. Injection given at nine P.M.: fever soon after subsided.

14th.—Improving. Fomentations omitted.

15th.—Has return of pain in head; eyes more injected; tongue brown and dry; pulse feeble; bowels not opened.

The warm fomentations to be renewed, and an enema to be administered.

Nine P.M.—Pain in the head removed; tongue moist; no fever.

16th.—Her feelings are better; a disposition to coma exists; the eyes are injected, and, when sleeping, they roll upwards under the lids half closed.

Fifty leeches behind the ears; and an irritating revulsive injection of Infusion of Jalap ʒj. Senna ℥ ij. Water ℔j.

Evening.—Bowels opened three times by injection, stools natural colour. Head very little relieved; tongue moist, but furred.

Sinapisms to ankles, and fomentations to be continued; hair to be removed from the head; and cold constantly applied during the night.

17th.—Much better: no disposition to drowsiness; eyes clear; no pain in head; tongue furred, but white and moist.

Ordered ℥ss. Castor-oil. Sago for diet.

18th.—Continues mending. Bowels opened by oil; tongue moist, fur cleaning off; no pain in epigastrium or abdomen; pulse and skin natural.

19th.—Entered into convalescence; diet increased.

*Observations.*—Riggins had been subjected to the perturbating practice, employed with great activity, but without the least improvement: it may be said, with an aggravation of the symptoms. When she entered the house, her condition was certainly most unfavorable, if not desperate, but a treatment calculated directly to diminish the inflammation existing in the stomach and meninges of the brain brought about an immediate change, and placed her in safety.

Case III.—Fleshower, aged about thirty-five, an assistant nurse in the women's clinical medical ward. In the fall she had intermittent fever, for which she had entered the house: it was accompanied with violent paroxysms of fever, headache, and gastric irritation. She was bled from the arm, and put on barley-water for sole diet. The intermittent disappeared after the third or fourth day of treatment. She had continued well, and was employed as an assistant in the ward until January 10th, when she was attacked with chill, pains in limbs, followed by intense headache, with pain in the chest. The bowels were bound; the tongue dry, furred, brown colour; nausea, restlessness, jactitation; epigastrium sensible, pressure occasioning acute pain; thirst ardent; eyes injected with blood; pulse full, frequent, tense; skin hot and dry.

Twelve ounces of blood were taken from the arm; an injection of Infus. Sennæ was directed; cups to head and epigastrium; blankets dipped in hot water to the legs; cold to the head; acidulated demulcent drinks taken cold.

January 11th.—Bowels opened by injection: pain in epigastrium increased; distress of patient greater; all the symptoms more unfavorable. Simple enema to be repeated, and sixty leeches to epigastrium: the leeches were applied at twelve o'clock.

Evening.—Better in every respect. Immediately after the application of the leeches, the headache, pain in epigastrium, restlessness and distress, were dissipated. The skin is now moist and of natural temperature; tongue moist and soft.

The hot fomentations to lower extremities continued. Diet, acidulated barley-water.

12th.—Improving. Diet continued. 13th.—Still better.

14th.—Committed an error of regimen: fever returned; tongue dry, furred; pain in side. Cups were directed, to be followed by a blister. Not experiencing relief, some hours after V.S. ℥xij. Absolute diet. 15th.—Better.

16th.—Breathing oppressed; pain returned.—V.S. ℥viij.

17th.—Better; pain continues, and pulse excited.—V.S. ℥x.; treatment, absolute diet. 18th.—Improving.

19th.—Symptoms have all disappeared. Increase diet.

20th.—Convalescent.

*Observations.*—In this case the patient came under treatment which rarely happens with the patients of this institution, from the commencement of the disease, and the physiological treatment could be brought into action against the simple disease, not aggravated by the effects of disturbing remedies on the organs. Cups were resorted to in the first instance, to economise the expense of leeching. I have always observed, however, that cups to the epigastrium, when it is very sensible and pain is acute on pressure, by the pain they occasion, aggravate the symptoms. This case is an instance of the kind. The effects of the leeches in reducing gastric irritation, were most conspicuously displayed in this case. It is also to be remarked that the cerebral symptoms were immediately relieved on the diminution of the gastric irritation by the leeching. This circumstance I have repeatedly observed. It is a strong proof that in fevers the cerebral disorder is sympathetic; and this effect may almost certainly be relied on, from local depletion from the epigastrium in fevers, when the continuance of the morbid sympathetic irritation has not become permanent in the brain or its meninges.

On the 14th, pleuritic symptoms were manifested, which easily yielded to a pure, unmixed antiphlogistic treatment.

The above cases are detailed by Dr. Jackson, in the American Journal of the Medical Sciences.

---

